# Preparation and Characterization of Cyperus-Derived Exosomes Loaded with Selenium Nanoparticles for Selenium Delivery Based on Exosome Protein Quantitation

**DOI:** 10.3390/foods14152724

**Published:** 2025-08-04

**Authors:** Dexiu Zhao, Xiaojun Yang, Abulimiti Kelimu, Bin Wu, Weicheng Hu, Hongbo Fan, Lei Jing, Dongmei Yang, Xinhong Huang

**Affiliations:** 1 College of Food and Pharmaceutical Science, Xinjiang Agricultural University, Urumqi 836500, China; dexiuzhao@163.com (D.Z.); abkaram@xjau.edu.cn (A.K.); jinglei1008@yeah.net (L.J.); ydm17690742872@163.com (D.Y.); 2 Institute of Agricultural Products Storage and Processing, Xinjiang Academy of Agricultural Sciences, Urumqi 836500, China; 42042615@qq.com; 3 Medical College, Yangzhou University, Yangzhou 225001, China; hu_weicheng@163.com; 4 College of Food Science and Engineering, South China University of Technology, Guangzhou 510641, China; 422488792@qq.com; 5 Xinjiang Camel Milk Engineering Technology Research Center, Altay 836500, China; huangxinhong0916@163.com

**Keywords:** SeNPs, exosome, protein quantitation, PEG precipitation, ultrasonic cell fragmentation

## Abstract

Appropriate carriers or templates are crucial for maintaining the stability, biological activity, and bioavailability of selenium nanoparticles (SeNPs). Selecting suitable templates remains challenging for fully utilizing SeNPs functionalities and developing applicable products. Exosome-like nanoparticles (ELNs) have gained importance in drug delivery systems, yet research on selenium products prepared using exosomes remains limited. To address this gap, we utilized Cyperus bean ELNs to deliver SeNPs, investigated three preparation methods for SeNPs-ELNs, identified the optimal approach, and performed characterization studies. Notably, all three methods successfully loaded SeNPs. Ultrasonic cell fragmentation is the optimal approach, achieving significant increases in selenium loading (5.59 ± 0.167 ng/μg), enlargement of particle size (431.17 ± 10.78 nm), and reduced absolute zeta potential (−4.1 ± 0.43 mV). Moreover, both exosome formulations demonstrated enhanced stability against aggregation during storage at 4 °C, while their stability varied with pH conditions. In vitro digestibility tests showed greater stability of SeNP-ELNs in digestive fluids compared to ELNs alone. Additionally, neither ELNs nor SeNP-ELNs exhibited cytotoxicity toward LO_2_ cells, and the relative erythrocyte hemolysis remained below 5% at protein concentrations of 2.5, 7.5, 15, 30, and 60 μg/mL. Overall, ultrasonic cell fragmentation effectively loaded plant-derived exosomes with nano-selenium at high capacity, presenting new opportunities for their use as functional components in food and pharmaceutical applications.

## 1. Introduction

As a toxic and essential trace element, selenium is considered insufficient when human intake is below 40 μg/d and becomes toxic beyond 40 μg/d [1]. Selenium has been intensively studied for its roles in antidiabetic, antioxidant, liver protective, lipid-lowering, and anticancer functions in recent years [2,3,4,5]. Since selenium cannot be synthesized within the body, it must be obtained through diet. Selenium nanoparticles, a simple, low-toxic form of selenium, exhibit greater efficacy than organic and inorganic forms [6]. However, in the absence of dispersion stabilizers or templates, selenium nanoparticles are susceptible to stratification, aggregation, and passivation, resulting in instability and a reduced ability to remain active [7]. Due to challenges in controlling selenium dosage and its potential toxicity, researchers are investigating suitable carriers or templates to develop selenium products that are low in toxicity, highly efficient, and stable.

**Figure 1:**
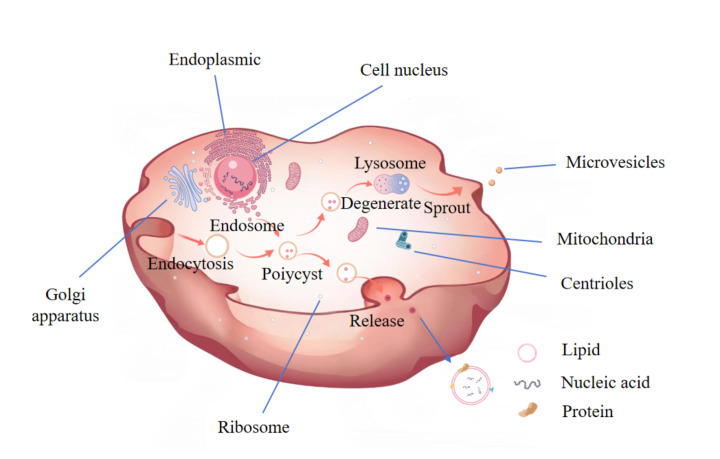
A Schematic representation of the process of exosome formation. This Figure is drawn by Medpeer.

As an extracellular vesicle, exosomes are secreted by cells and contain a variety of bioactive molecules such as proteins, nucleic acids, and lipids [8]. Their formation mechanism involves the creation of early intracellular bodies following cell membrane endocytosis, which encapsulates certain proteins and RNA within the cell, leading to the formation of multivesicular bodies. These bodies then fuse with the cell membrane, releasing exosomes outside the cell to facilitate communication between various cells [9] (Figure 1). Nearly all cell types, including those found in blood, urine, cerebrospinal fluid, and plants, can secrete exosomes [10,11]. Recently, exosomes have gained significant attention for their crucial role in intercellular communication, disease occurrence, and treatment [9]. Exosomes can either merge with the membrane of target cells or impact recipient cells through endocytosis [12]. The phospholipid bilayer of exosomal vesicles shields internal biomolecules from enzymes present in body fluids, thus preserving their integrity and biological activity. Notably, exosomal RNA is resistant to trypsin, as previously reported [12]. Additionally, the specialized glycoproteins enveloping milk exosomes and the surface membrane proteins make them resistant to pepsin and trypsin, allowing exosomes to withstand the digestive tract [13]. Hence, they serve as carriers for drugs or other unstable functional components, ensuring the stability, biological activity, and bioavailability of the encapsulated materials [14,15]. Current research predominantly remains within the realm of clinical medicine and recipient cells [16,17], focusing largely on animal cell exosomes, which are costly. In contrast, plant-derived exosomes offer a broadly available, resource-rich, and cost-effective alternative.

Known as oil sedge, tiger nuts, underground walnut, underground chestnut, and ginseng fruit, Cyperus beans exhibit physiological functions such as lowering blood lipids, reducing blood sugar, antioxidant effects, and liver protection [18,19]. Notably, both grapefruit exosomes and naringin in grapefruit exhibit antitumor effects. Similarly, ginger exosomes show anti-inflammatory activity like ginger itself. Exosomes derived from different plants demonstrate activities similar to their source plants [20]. In contrast, plant-derived exosomes are more accessible, cost-effective, safe, and suitable for oral delivery of biological drugs. Given the need for developing new resources and the physiological functions of Cyperus, Cyperus is chosen as the exosome source, potentially allowing the exosomes to exhibit relevant physiological activities as delivery carriers or to display synergistic antioxidant effects with loaded selenium nanoparticles.

Various methods exist for extracting exosomes, including ultracentrifugation, density gradient centrifugation, chromatography sorting, ultrafiltration, magnetic bead capture, and polyethylene glycol (PEG) precipitation [21,22,23]. The extraction of exosomes via PEG precipitation is straightforward, conserving material resources and more apt for mass production. Typically, the sample solution containing exosomes is mixed with equal volumes of PEG solution and co-incubated overnight at 4 °C. Excess PEG is then removed, and exosome precipitation is collected by centrifugation [24].

**Figure 2:**
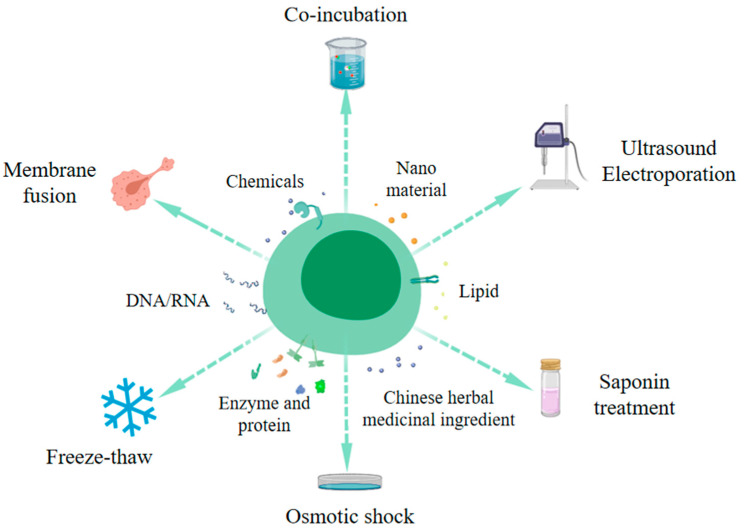
Schematic diagram of the method of exosome engineering modification. (This figure is drawn by Medpeer).

Exosomes are widely utilized in the delivery of drug molecules and can be loaded with nucleic acids, proteins, lipids, enzymes, nanomaterials, and drugs. Several methods exist for exosome modification, including osmotic shock, liposome fusion, freeze–thaw, electroporation, ultrasound, saponin treatment, and co-incubation [25,26,27] (Figure 2). The ultrasound method is employed to create transient pores in the plasma membrane, increasing membrane permeability and enabling the entry of target molecules. This method is notably efficient and accommodates both hydrophobic and hydrophilic substances.

With advancements in modern medicine, more people are receiving timely and effective treatment; however, chronic diseases remain a challenge. Although drugs are available for treating chronic conditions, they often come with side effects. Using dietary management as an adjuvant treatment can often yield positive results. Thus, the aim of this study was to combine SeNPs with ELNs for oral delivery. Despite the biological importance of ELNs, their application in combination with SeNPs as a dietary intervention strategy is still in its early stages. Therefore, the primary objective of this study was to isolate specific exosome-like nanovesicles from Cyperus beans using PEG precipitation and to further investigate the effects of different incorporation methods on SeNPs loading. Additionally, we examined the effects of different pH levels, storage temperatures, and in vitro digestibility on particle size, polydispersity index (PDI), and zeta potential. Furthermore, we conducted studies on cytotoxicity and erythrocyte compatibility.

This study presents two novel advancements that collectively redefine the potential of plant-derived exosomes as nanocarriers. First, we describe the initial isolation and comprehensive characterization of ELNs derived from Cyperus beans. By establishing a PEG precipitation–ultracentrifugation protocol, we provide the first physicochemical profile of Cyperus-derived ELNs, previously unreported in the literature. Second, to overcome the persistent challenge of loading inorganic nanoparticles into ELNs, we developed and validated an ultrasound-triggered in situ synthesis method. A controlled primary ultrasound pulse transiently permeabilizes the ELNs membranes, enabling diffusion of sodium selenite (Na_2_SeO_3_) into the vesicular lumen. A subsequent secondary pulse delivers ascorbic acid into the same compartment, inducing intravesicular generation of SeNPs. Crucially, this dual-pulse methodology achieves significantly higher SeNP loading efficiency than conventional ultrasonic baths or passive incubation methods.

## 2. Materials and Methods

### 2.1. Materials and Reagents

Phosphate buffered saline (PBS, pH = 7.2, 0.01 M), (Guangzhou Hewei Pharmaceutical Technology Co., Ltd., Guangzhou, China); Polyethylene glycol 6000 (PEG6000, molecular weight 5500–7000), (Shanghai Shanpu Chemical Co., Ltd., Shanghai, China); BCA protein kit (Keyizhe Electromechanical Engineering Co., Ltd., Beijing, China); sodium selenite (Na_2_SeO_3_), (Tianjin Beilian Fine Chemical Development Co., Ltd., Tianjin, China); ascorbic acid (Tianjin Beilian Fine Chemical Development Co., Ltd., Tianjin, China); artificial gastric juice and intestinal juice (Dongguan Chuangfeng Automation Technology Co., Ltd., Dongguan, China); the CCK-8 kit (Wuhan Feen Biotechnology Co., Ltd., Wuhan, China); LO_2_ Cell (Shanghai Enzyme Research Biotechnology Co., LTD, Shanghai, China).

### 2.2. Synthesis of SeNPs

Ascorbic acid reduces and undergoes a redox reaction with Na_2_SeO_3_, converting the positively charged tetravalent selenium in sodium selenite to elemental selenium [28,29]. The reaction Formula (1) is as follows:

(1) Na_2_SeO_3_+2C_6_H_8_O_6_→Se↓+2C_6_H_6_O_6_+3H_2_O

**Figure 3:**
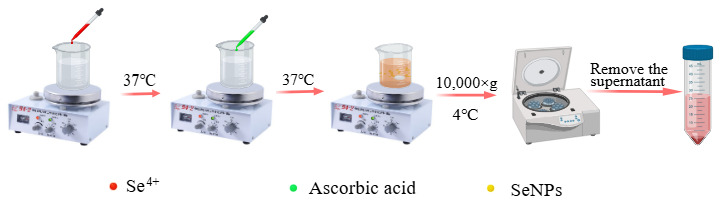
Schematic diagram of the synthesis of SeNPs without a template.

A sodium selenite solution (0.1 M) was magnetically stirred in a thermostatic water bath (DF-101S, Shanghai Yixin Scientific Instrument Co., Ltd., Shanghai, China) at 37 °C, followed by the addition of ascorbic acid solution (0.8 M). The reaction mixture was protected from light and allowed to proceed for 2 h [30,31]. The pH of the solution was adjusted to 7.0 using NaOH or HCl [32]. During the reaction, the solution gradually changed from transparent to orange and finally to dark red [33]. After the reaction, excess ascorbic acid was removed by centrifugation (TGL-16M, Hunan XiangXin Instrument Co., Ltd., Yueyang, China) at 10,000 g for 2 h at 4 °C, resulting in SeNPs precipitation [34] (Figure 3).

### 2.3. Extraction of Cyperus ELNs

The beans were ground and then passed through a 100-mesh sieve. The ground powder was mixed with PBS buffer at a 1:3 ratio in a centrifuge tube and centrifuged at 3000 r/min for 30 min to remove the precipitate. The obtained supernatant was centrifuged again at 10,000 r/min for 30 min to remove any remaining precipitates. The resultant supernatant was filtered to remove impurities and blended with PEG6000 solution at a volume ratio of 1:1 and stored at 4 °C overnight [24,35,36].

**Figure 4:**
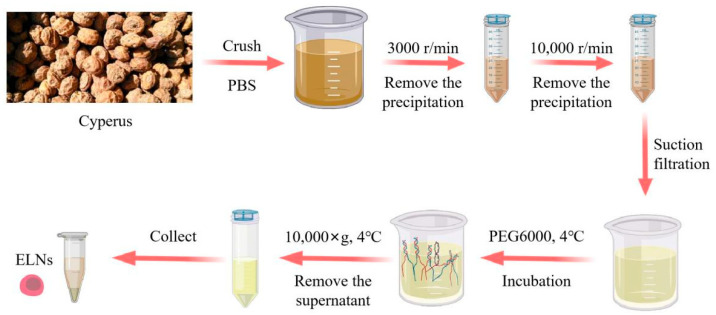
Schematic diagram of the exosome extraction process by PEG precipitation.

After storage, the mixed solution was centrifuged at 10,000 g for 30 min at 4 °C, and the supernatant was removed. Upon being suspended in 1 mL of PBS, the precipitate was centrifuged at 10,000 g for 30 min, and the Cyperus ELNs were obtained after repeating the above precipitation and suspension process three times [37] (Figure 4).

### 2.4. SeNPs-ELNs Preparation

#### 2.4.1. Preparation by Ultrasonic Washer

**Figure 5:**
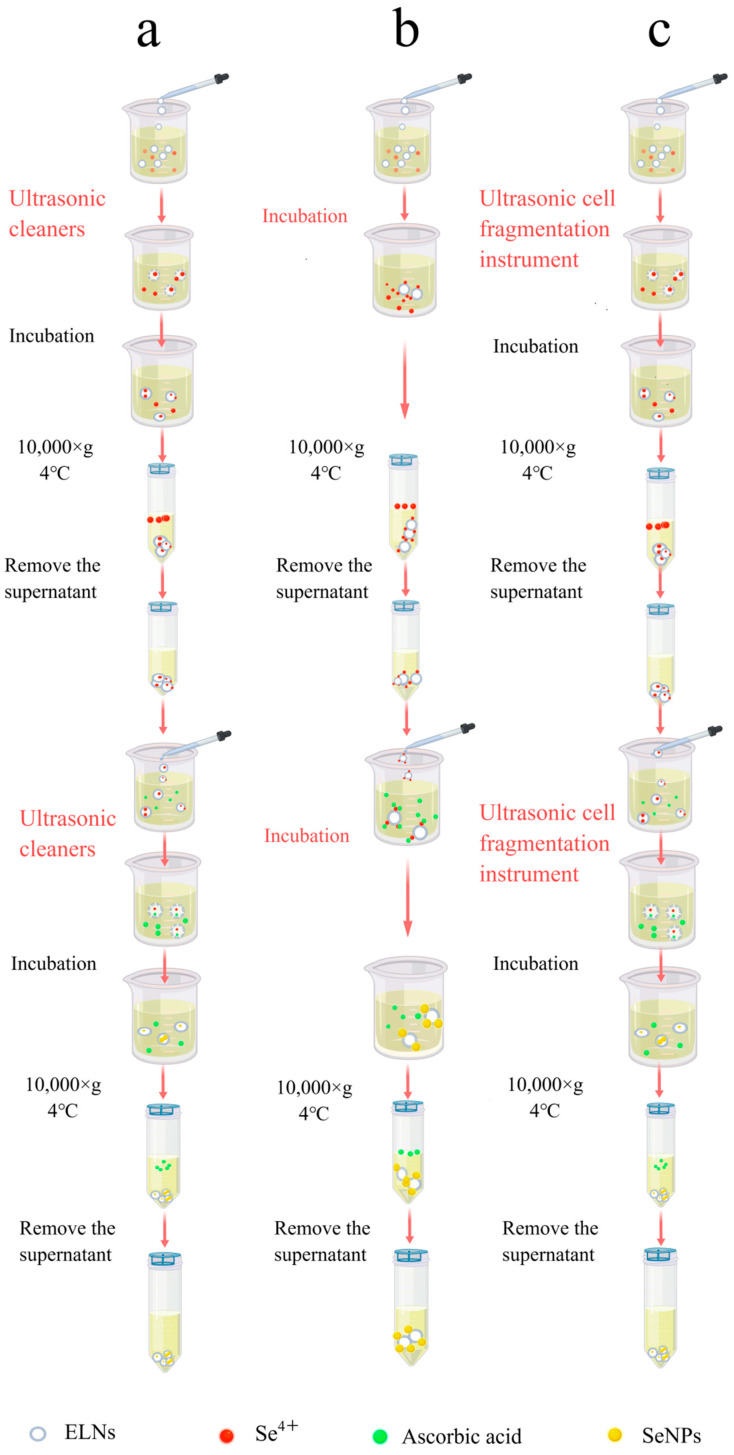
(**a**) Schematic diagram of the process for preparing SeNPs-ELNs I by ultrasonic washer. (**b**) Schematic diagram of the process for preparation of SeNPs-ELNs II by incubation. (**c**) Schematic diagram of the process for preparing SeNPs-ELNs III using an ultrasonic cell fragmentation instrument.

SeNPs-ELNs I: After mixing the exosome suspension with Na_2_SeO_3_ solution (0.1 M) and stirring thoroughly at room temperature, the Na_2_SeO_3_ was incorporated into the exosomes using an ultrasonic cleaner (KQ5200DE, Kunshan Ultrasonic Instrument Co., Ltd., Kunshan, China) at 50% amplitude (100 W) at 37 °C for 5 min. The mixture was then incubated at 37 °C for 60 min to reconstitute the membrane. The surplus Na_2_SeO_3_ was removed by centrifugation (10,000× *g*, 4 °C). The precipitate was collected and resuspended in PBS buffer. This process was repeated several times until the supernatant’s color remained unchanged upon addition to the ascorbic acid solution. The final precipitate was resuspended in 1 mL of PBS and combined with ascorbic acid solution (0.8 M) by stirring thoroughly. The Se^4+^ within exosomes reacted with the ascorbic acid in an ultrasonic washer (100 W, 37 °C) for 5 min, and the mixture was then incubated at 37 °C for 60 min to reconstitute the exosome membrane. The excess ascorbic acid was removed by centrifugation (10,000× *g*, 4 °C) for 30 min, and the SeNPs-ELNs were obtained after repeating the precipitation and suspension process three times (Figure 5a).

#### 2.4.2. Preparation by Incubation

SeNPs-ELNs II: The exosome solution was mixed with a Na_2_SeO_3_ solution (0.1 M), stirred thoroughly at room temperature, and incubated at 37 °C for 12 h to bind Se^4+^ to the exosome membrane [38]. The excess Na_2_SeO_3_ was removed by centrifugation (10,000× *g*, 4 °C) for 30 min, and the precipitate was resuspended in PBS buffer. Centrifugation was repeated several times until the supernatant color did not change when added to the ascorbic acid solution.

The Na_2_SeO_3_-loaded exosome solution was then added to an ascorbic acid solution (0.8 M), stirred thoroughly, and incubated at 37 °C for 12 h to enable the ascorbic acid to bind to the exosome membrane. Subsequently, Se^4+^ and ascorbic acid reacted to form SeNPs. The excess unreacted ascorbic acid was removed using the procedures mentioned above (Figure 5b).

#### 2.4.3. Preparation by Ultrasonic Cell Fragmentation Instrument

SeNPs-ELNs III: After mixing the exosome solution with a Na_2_SeO_3_ solution (0.1 M) at room temperature, Na_2_SeO_3_ was loaded into the exosomes using an ultrasonic cell fragmentation instrument (WM-650, Shanghai Micro Mi Sonication Co., Ltd., Shanghai, China) at 15% amplitude (97.5 W) for 3 min with a pulse interval of 10:5. The mixture was then incubated at 37 °C for 8 h to reconstitute the membrane. The excess Na_2_SeO_3_ was removed by centrifugation (10,000× *g*, 4 °C), the precipitate was collected, resuspended in PBS buffer, and repeatedly centrifuged until the supernatant’s color no longer changed when added to the ascorbic acid solution. The Na_2_SeO_3_-loaded exosome solution was then added to ascorbic acid solution (0.8 M), stirred thoroughly, and the ascorbic acid was loaded into exosomes using the same ultrasonic settings. The mixture was then incubated at 37 °C for 8 h to reconstitute the membrane [26,39]. The excess unreacted ascorbic acid was removed using the procedures described previously (Figure 5c).

### 2.5. The Fourier Transform Infrared Spectrum (FTIR)

FTIR is generally used to analyze intermolecular interactions. The ELNs and SeNPs-ELNs were freeze-dried. Lyophilization was performed using a freeze-dryer (YTLG-12D-80, Shanghai Yetuo Technology Co., Ltd., Shanghai, China) under the following protocol: samples were pre-frozen at −80 °C for 12 h, followed by primary drying at −40 °Cand 10 Pa for 24 h, and secondary drying at 25 °C and <5 Pa for 6 h. Then the dried samples were mixed with dried potassium bromide in a mass ratio of 1:100. The mixture was then ground repeatedly to form transparent potassium bromide tablets. The FTIR (USA-Thermo Fei-Nicolet IS5, Zhongke Ruijie Technology Co., Ltd., Beijing, China) running parameters were set as follows: scan range 400–4000 cm^−1^, resolution 4 cm^−1^, and scan 32 times [40,41].

### 2.6. The UV-Visible Light Spectrum (UV-Vis)

UV-Vis spectroscopy analysis was employed to investigate intermolecular interactions, performed by dispersing nanoparticles in an aqueous solution. The SeNPs, ELNs, and three SeNPs-ELNs were analyzed using a UV-vis spectrophotometer (TUV765, Shanghai Youke Instrument Co., Ltd., Shanghai, China), with a wavelength scan range of 200–400 nm and a scan resolution of 1 nm [42,43,44].

### 2.7. Transmission Electron Microscopy and Energy Dispersive X-Ray Spectroscopy (TEM-EDS Mapping)

TEM-EDS Mapping was utilized to determine the distribution and content of elements, serving as direct evidence for the presence of these elements. The elemental composition was assessed using energy-dispersive X-ray spectroscopy (EDS) combined with a transmission electron microscope (JEM-F200, JEOL Ltd., Tokyo, Japan). N, O, and Se elements from SeNPs, ELNs, and three SeNPs-ELNs were analyzed at an accelerating voltage of 200 kV, with results reported as percentages of atomic weight [31].

### 2.8. X-Ray Photoelectron Spectroscopy (XPS)

XPS analysis was conducted using the Thermo Scientific ESCALAB 250Xi (Thermo Fisher Scientific, Waltham, MA, USA) to assess the valence states of elements. After vacuum freeze-drying the samples of SeNPs, ELNs, and three SeNPs-ELNs (ZX-LGJ-27, Shanghai Zhixin Experimental Instrument Technology Co., Ltd., Shanghai, China), lyophilized powders were analyzed. The chamber vacuum during analysis was maintained at 5 × 10^−9^ torr, using a monochromatic Al Kα source (Mono AL Ka) with an energy of 1486.6 eV. The test area was 500 μm, and the scan mode was CAE. Full-spectrum Scans were performed with an energy of 100.0 eV and a step of 1.00 eV; narrow-spectrum scans were conducted at 20 eV with a step of 0.05 eV. The number of scans was set to 1, and all resulting spectra were calibrated using C1s at 284.6 eV [45].

### 2.9. X-Ray Diffraction (XRD)

Post vacuum freeze-drying, the samples of SeNPs, ELNs, and three SeNPs-ELNs were prepared for XRD analysis (Bruker D8 Advance, Brooke Technology, Ltd., West Bend, WI, USA). The samples were placed into the sample holder, leveled, and scanned. The selected working voltage and current were 40 kV and 40 mA, respectively, with a scanning range from 5° to 90°. The scanning mode was continuous step scanning with a step size of 0.02519 and a scanning speed of 10 degrees per minute [42,44,46,47].

### 2.10. Scanning Electron Microscope (SEM)

An appropriate amount of lyophilized powder was placed on the copper conductive adhesive on the carrier and coated with gold [29]. The set voltage was 5.00 kV, and the samples were examined under a SEM (Germany ZEISS SIGMA HD, Shanghai Diguan Industrial Co., Ltd., Shanghai, China) at 20 k× and 50 k× magnifications.

### 2.11. Determination of Loading Amount

Load volume (LC) is defined as the target molecule mass per unit mass carrier. Such as the target molecule ng/μg exosomes, pM/μg exosomes. The calculation Formula (2) is as follows:

(2) LC = loaded quantity of cargo (mass, moles)/input of exosome (mass, particle)

The loaded cargo, specifically the content of SeNPs, was determined primarily using the hydride atomic fluorescence spectrometer (AFS-9600, Shanghai Analysis Instrument Co., Ltd., Shanghai, China) as per GB 5009.93–2017 “Determination of Selenium in National Standard for Food Safety” [48]. Samples were digested in a mixture of HNO_3_ and HClO_4_. Upon the solution becoming clear, colorless, and emitting white smoke, it was heated until the volume reduced to 2 mL, then cooled. Subsequently, 5 mL of 50% HCl was added, and the solution was again reduced to 2 mL. After cooling, an additional 5 mL of HCl was added, and the volume was adjusted to 50 mL with ultrapure water for measurement via peak area [49]. The testing parameters were set as follows: negative high voltage of 300 V; lamp current of 80 A; shield gas flow of 800 mL/min; carrier gas flow of 300 mL/min; heater temperature of 200 °C; atomization height of 8 mm.

The input amount of exosomes was measured using the Bicinchoninic Acid (BCA) protein assay kit to determine protein concentrations [50,51]: An appropriate amount of Reagent A and Reagent B was mixed in a 50:1 (*v*/*v*) ratio to prepare the BCA working solution. After configuring a gradient concentration solution of Bovine Serum Albumin (BSA) Protein Standard, 100 μL of each sample and BSA Protein Standard solution were mixed with the working solution, transferred to a colorimetric cup, incubated at 37 °C for 30 min, and allowed to cool to room temperature. The absorbance at 562 nm was measured using a spectrophotometer. The protein concentration of the sample was calculated based on the protein standard concentration and absorbance values.

### 2.12. Transmission Electron Microscopy (TEM)

ELNs and SeNPs-ELNs were examined using TEM (JEM-1230, JEOL, Tokyo, Japan) [52]. The procedure involved [53]: dispersing the samples by sonication for 5 min, placing them on copper grids, and allowing them to rest at room temperature for 15 min. Negative staining was then applied for 1 min using a 2% aqueous phosphotungstic acid solution, followed by blotting away the excess dye with filter paper. The copper grids were dried under incandescent lamps and imaged at 80 kV using a TEM system [54,55].

### 2.13. Dynamic Light Scattering (DLS)

Particle size and distribution were analyzed using a Malvern laser scattering particle size meter (Malvern Zetasizer Nano S90, Beijing Yonaka Technology Co., Ltd., Beijing, China). This method observes changes in scattered light over time, based on the random Brownian motion of the nanoparticles. The Stokes–Einstein equation was used to determine particle size, polydispersity index (PDI), and Zeta potential for ELNs and SeNPs-ELNs III [56]. Before testing, the DLS system was warmed up for 30 min. Samples were thoroughly shaken to ensure uniformity. One milliliter of the suspension was diluted with deionized water, placed in the sample pool, and analyzed using Zetasizer software v7.13. Instrument settings included a temperature of 25 °C, an equilibration time of 70 s, a refractive index of 1.330, and a count rate of 100–200 kcps [57]. Every sample was measured three times to calculate the size, distribution, and Zeta potential.

### 2.14. Temperature Stability Investigation

The suspension of the ELNs and SeNPs-ELNs III was stored at 25 °C, 4 °C, and −20 °C for 14 days [58,59,60], and the particle size, PDI, and Zeta potential of the suspension were determined [38]. The operation steps and conditions refer to 2.13.

### 2.15. pH Stability Investigation

The pH of the suspension of ELNs and SeNPs-ELNs III was adjusted to 3, 4, 5, 6, 7, and 8 with 1 M NaOH or HCl. The measurements were carried out after incubation for 24 h at 25 °C [40]. The operation steps and conditions refer to 2.13.

### 2.16. Digestive Stability Test

Digestive stability tests for ELNs and SeNPs-ELNs III were performed in PBS buffer, simulated gastric fluid (SGF), and simulated intestinal fluid (SIF), following the manufacturer’s instructions. SGF mainly contains NaCl, HCl, and pepsins, while SIF contains phosphate and trypsin. Suspensions of the ELNs and SeNPs-ELNs III were mixed with PBS, SGF, and SIF in a 1:20 volume ratio and incubated at room temperature. Samples were collected after 2 h (SGF) and 7 h (SIF) to measure particle size, PDI, and Zeta potential, using the methods described in Section 2.13.

### 2.17. Cytotoxicity Measurement

The Cell Counting Kit-8 (CCK-8) assay was employed to evaluate the cytotoxic effects of ELNs and SeNPs-ELNs III on LO_2_ cells. Cells in the logarithmic growth phase were utilized for the experiments [61]. Three groups were established according to the kit instructions: the blank group, consisting of medium and CCK-8; the control group, comprising cells, medium, and CCK-8; and the experimental group, which included cells, medium, ELNs, and SeNPs-ELNs III (100 μL/well, with protein concentrations of 2.5, 7.5, 15, 30, and 60 μg/mL). Subsequently, 10 μL of CCK-8 solution was added to each well and incubated at 37 °C in 5% CO_2_ for 2 h. Each group had three replicates. Absorbance values were measured using a microplate reader (Varioskan LUX, Thermo Fisher SCIENTIFIC, Waltham, MA, USA) at 450 nm, and cell viability was calculated using Equation (3) [32,62,63].

(3) Cell viability(%) = [(A_s_ − A_b_)/(A_c_ − A_b_)] × 100

where A_s_, A_b_ and A_c_ are the absorbance of the experimental, blank, and control group, respectively.

### 2.18. Relative Erythrocyte Hemolysis Rate Study

ELNs and SeNPs-ELNs III were diluted with PBS to achieve concentrations of 2.5, 7.5, 15, 30, and 60 μg/mL. A 1 mL volume of each concentration was placed into 1.5 mL centrifuge tubes. A healthy volunteer (male, 32 years old) was recruited to provide 2 mL of peripheral blood, collected using vacuum venipuncture; the anticoagulated whole blood was then centrifuged at 1000 rpm for 10 min. From this, 0.2 mL of the lower layer of red blood cells was added to each 1.5 mL centrifuge tube, followed by 1 mL of saline, and the mixture was gently inverted and centrifuged at 1000 rpm for 10 min. The supernatant was carefully aspirated, and 0.25 mL of saline was added to the red blood cells for dilution. Subsequently, 20 μL of the red blood cell suspension was added to the test sample, 20 μL to 1 mL of PBS as a negative control, and 20 μL to 1 mL of deionized water as a positive control. These were incubated at 37 °C with rotation for 0.5 h; both sample and control setups were replicated three times. After incubation, samples were centrifuged at 3500 rpm (about 1000 g) for 5 min. Then, 0.2 mL of the supernatant from each sample was transferred to a 96-well plate. The absorbance at 545 nm was measured using a microplate reader, and the relative erythrocyte hemolysis rate was calculated using Formula (4) [64]:

(4) Hemolysis(%) = [(D_t_ − D_nc_)/(D_pc_ − D_nc_ )] × 100%

where D_t_, D_nc,_ and D_pc_ are the absorbance of the experimental sample, negative control group, and positive control group, respectively. (The hemolysis rate of red blood cells is less than 5%, indicating that the material meets the requirements of medical materials and does not cause serious hemolysis reaction; the hemolysis rate is higher than 5%, indicating that the material will cause hemolysis reaction.)

### 2.19. Statistical Analyses

All measurements were performed in triplicate. Statistical analyses were carried out using SPSS statistical software (SPSS 18.0, Chicago, IL, USA). One-way Analysis of Variance (ANOVA) was applied to analyze the data statistically. The difference between mean values was determined by the Pair-Sample *t*-Test at an α-level of Statistical analysis 5%.

## 3. Results and Discussion

### 3.1. FTIR Analysis

**Figure 6:**
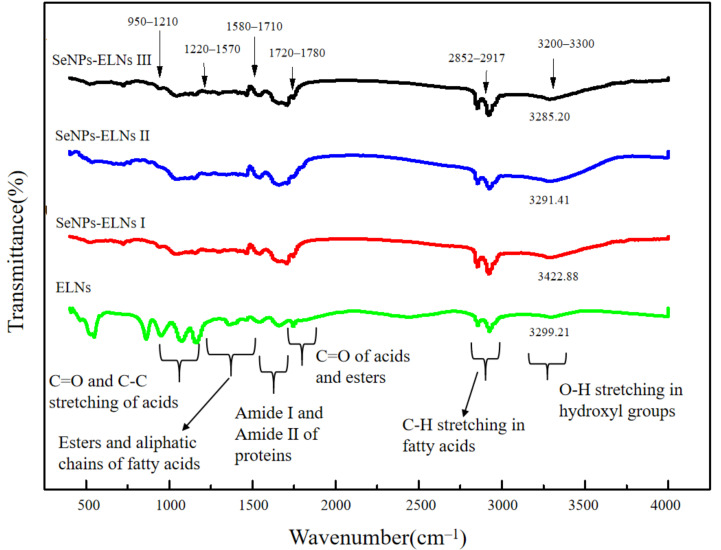
The Fourier transform infrared spectroscopy of ELNs and SeNPs-ELNs prepared by different methods.

Even though there are several investigations on uploading active components into exosomes [65,66], to the best of our knowledge, incorporation of SeNPs into Cyperus-derived exosomes is yet to be reported. ELNs were isolated from Cyperus, and SeNPs-ELNs were prepared by ultrasonic washer, incubation, and ultrasonic cell fragmentation methods. Differences in characteristic Fourier transform infrared spectroscopy absorption peaks are useful evidence to reflect the interactions between substances of a composite [67]. The absorption peaks of ELNs and three different preparations of SeNPs-ELNs through FTIR were measured to verify the interaction between the ELNs and SeNPs. As shown in Figure 6, the peaks of ELNs exhibited characteristic peaks in the range of 3300–950 cm^−1^, among which the main characteristic peaks at 3300–3200 cm^−1^, 2917–2852 cm^−1^, 1780–1720 cm^−1^, 1710–1580 cm^−1^, 1570–1220 cm^−1^, and at 1210–950 cm^−1^ were associated with O–H stretching, CH stretching of fatty acids, C=O of acids and esters, amide I and amide II of proteins, esters and aliphatic chains of fatty acids, and C=O and C-O stretching of acids, respectively. Compared with ELNs peaks, the FTIR spectra of SeNPs-ELNs exhibited several differences, especially in peak transmittance, as well as weak frequency shifts at 3300–3200 cm^−1^ and at 1710–1580 cm^−1^, which might be the result of electrostatic interaction between SeNPs and hydroxyl groups of polysaccharides and amino groups of proteins [68].

Peak shifts within the 1580–1780 cm^−1^ range indicate altered electronic environments of carbonyl groups, suggesting direct interactions between C=O oxygen atoms (in lipids, fatty acids, esters, or membrane proteins) and SeNPs surfaces through coordination or hydrogen bonding. Concurrently, changes in C-O stretching vibrations (1200–1300 cm^−1^, overlapping C=O in the fingerprint region) further indicate interactions with lipid polar groups or ester bonds. These variations, coupled with significant O-H band shifts, provide strong evidence for hydrogen bonding between ELNs’ surface hydroxyl groups (-OH in glycolipids/glycoproteins) and SeNPs. Shifts in amide I and amide II bands confirm SeNPs interactions with surface proteins, while C-H stretching vibrations (2850–2920 cm^−1^) indicate altered conformational dynamics of lipid fatty chains.

Collectively, these spectral changes—particularly pronounced shifts in C=O, C-O, and O-H vibrations—serve as definitive evidence of chemical interactions between SeNPs and ELNs’ biomolecular components (lipids, proteins, carbohydrates) at their interface. Consequently, the FTIR data demonstrate that SeNPs undergo specific chemical integration—rather than mere adsorption—within the ELNs’ structure. This validates the successful synthesis of the SeNPs-ELNs.

### 3.2. UV-Vis Analysis

**Figure 7:**
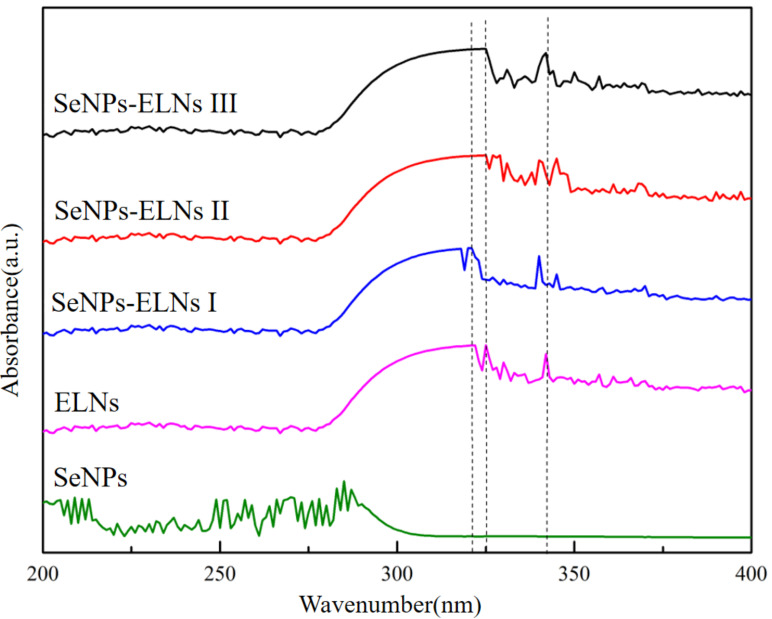
The UV-visible spectra of SeNPs, ELNs, and SeNPs-ELNs prepared by different methods.

The curve of SeNPs in Figure 7 indicates significant absorption between 250 and 300 nm, with broad absorption peaks and poor symmetry, reflecting an uneven particle size distribution, similar to findings in many previous studies [32,46,69]. ELNs and the three SeNPs-ELNs did not form distinct peaks due to their low selenium content and minimal light absorption values. Additionally, the introduction of ELNs as templates introduced heteroatom unsaturated groups such as (C=O), which caused n→π* electron transitions [70]. Moreover, the characteristic absorption peaks of the three SeNPs-ELNs shifted relative to those of the ELNs.

### 3.3. TEM-EDS Mapping Analysis

**Figure 8:**
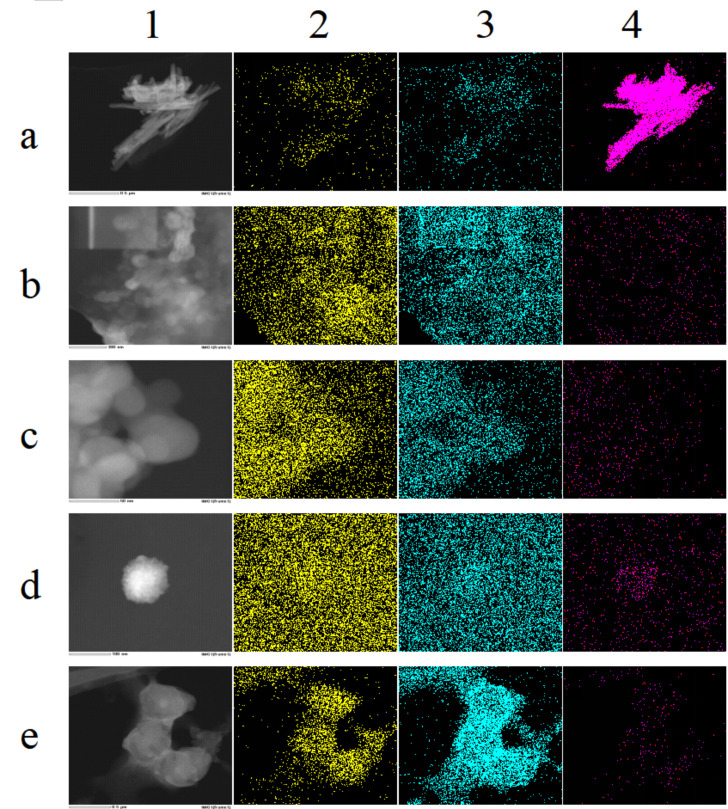
(**a**–**e**) correspond to SeNPs, ELNs, SeNPs-ELNs I, SeNPs-ELNs II, and SeNPs-ELNs III, respectively; (**1**–**4**) represent the TEM image, N distribution, O distribution, and Se distribution, respectively.

As depicted in Figure 8(a1), the TEM map of SeNPs illustrates stacked selenium lines [31], indicating that in the absence of a template, SeNPs are prone to aggregation. Figure 8(a2–a4), Figure 8a from TEM-EDS diagram shows that the surface of SeNPs contains Se, O, and N elements. The O element originates from the oxidation product of ascorbic acid; the surface percentage of Se elements is 83.46% in Table 1, confirming high selenium content and selenium as the primary component [28,71].

As shown in Figure 8(b1), ELNs exhibit poor dispersion and are prone to agglomeration. In the upper left corner of Figure 8(b1), the local magnification of individual particles is insufficient for selection for TEM-EDS Mapping analysis. Figure 8b indicates that the ELNs contain N and O elements, with the O element comprising 73.57% (Table 1), suggesting that oxygen is the predominant component. The nitrogen content is 26.43%, likely originating from the proteins present in the ELNs. In Figure 8(b4), the fluorescence distribution map of selenium shows a faint and sparse red fluorescence signal within the sample area. Table 1 indicates that the Se element of ELNs was not detected (ND), although an absorption peak for selenium is present in the spectrum of Figure 5b. This suggests that the selenium content may be below the detection limit of the instrument, exceeding its sensitivity range.

As shown in Figure 8(c2,c3), SeNPs-ELNs I contain N and O elements. The O element content is 99.21%, compared with 73.57% in the template, which may be attributed to the oxidation of ascorbic acid, indicating that sodium selenite is reduced. The N element content is significantly reduced to 0.79%, likely due to the disruption of membrane proteins in exosomes by the ultrasonic washer [55,72]. Figure 8(c4), the fluorescence distribution map of Se, displays a weakly sparse red fluorescence distribution. Although the Se element is ND, indicating very low Se content beyond the instrument’s detection range, an absorption peak of Se is observed in the spectrum (Supplementary Figure S1c).

As shown in Figure 8(d1), SeNPs-ELNs II burn out during scanning at 200 kV, similar to the region in the upper left corner of Figure 8(a1), which shows the template ELNs. This suggests that the voltage is too high or that the stability of these two biological samples is poor. Additionally, the surface contains elements such as N, O, and Se, with Se displaying a relatively aggregated red fluorescence distribution in the sample area (Figure 8(d4)). Moreover, Table 1 indicates that the Se element content of SeNPs-ELNs II is 0.17%, which is a significant increase compared to the template ELNs and the SeNPs-ELNs I.

SeNPs-ELNs III also contain N, O, and Se (Figure 8e). Compared with the 26.43% nitrogen content of the ELNs, the nitrogen content of the SeNPs-ELNs III is 4.48%, which may be due to the ultrasonic process disrupting the exosome membrane proteins. However, the nitrogen content is higher than that of SeNPs-ELNs I, suggesting that the ultrasonic cell fragmentation instrumentation causes less damage to the exosome membrane proteins. Furthermore, Se exhibits a relatively aggregated red fluorescence distribution in the sample region (Figure 8(e4)), and Table 1 shows that the Se element content of SeNPs-ELNs III is 1.72%, a substantial improvement compared to the ultrasonic washer and incubation methods. Based on selenium content, the ultrasonic cell fragmentation instrumentation method may be the optimal preparation technique.

### 3.4. XPS Analysis

**Figure 9:**
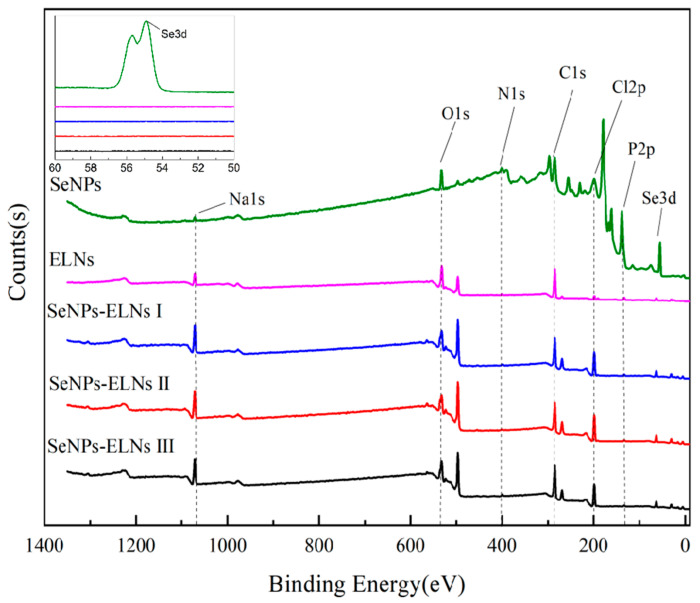
Full spectrum and selenium high-resolution map of SeNPs, ELNs, and three SeNPs-ELNs. Five black dashed lines represent the peaks of the O1s, N1s, C1s, Cl2p, and P2p orbitals, respectively.

In the initial phase of this study, the orange-red appearance of SeNPs and three SeNPs-ELNs led to a preliminary identification of the selenium as zero-valent. To precisely determine the valence states of selenium, XPS measurements were performed on SeNPs, ELNs, and three SeNPs-ELNs, with the binding energy results depicted in Figure 9. The analysis revealed that the materials primarily contain elements C, N, O, Na, Cl, P, and Se. Na, Cl, and P originated from NaCl, Na_2_HPO_4_, and NaH_2_PO_4_ in PBS buffer. According to the NIST X-Ray Photoelectron Spectrum Database–Standard Reference Database 20 (version 4.1), peak positions of Se electron binding energy at 55.0 and 55.1 correspond to zero-valent Se, indicating that the reaction producing zero-valent selenium from sodium selenite and ascorbic acid is viable [29]. It is inferred that the selenium in SeNPs-ELNs prepared by the three methods is also zero-valent. However, light intensity values were not detected in the Se 3d tracks of the three SeNPs-ELNs, likely due to the low content of zero-valent selenium not reaching the instrument’s detection limit. Furthermore, no characteristic electron binding energy peaks of higher-valent Se were observed in the three SeNPs-ELNs, suggesting that the loaded Se^4+^ has been fully reduced, or any unreduced Se^4+^ was removed by centrifugation [69] (Figure 9).

### 3.5. XRD Analysis

**Figure 10:**
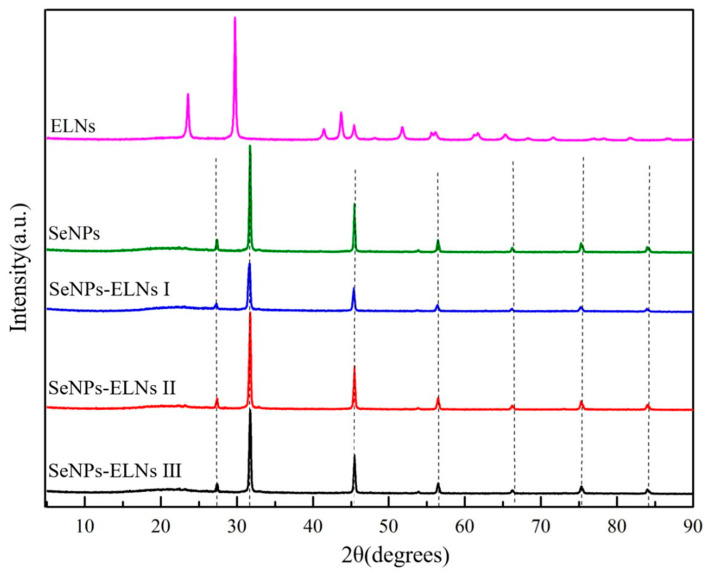
XRD spectrogram of SeNPs, ELNs, and three SeNPs-ELNs. The black dashed lines represent diffraction peaks from components in the PBS buffer, including Na_2_HPO_4_ and different crystal planes of NaCl.

The biological activity of SeNPs is influenced by their zero-valent state and amorphous structure, where the red amorphous SeNPs exhibit strong antioxidant and anticancer properties [71]. Elemental selenium features six allotropes: amorphous, glassy state, and monoclinic crystal system (α-, β-, γ-) along with the tripartite crystal system (t-Se) [73]. Based on the orange-red appearance and the XPS results, the three SeNPs-ELNs are likely zero-valent selenium. To verify their crystal form, XRD spectra of the samples were compared, with results shown in Figure 10. When matched against JCPDS cards, the sharp and narrow absorption peak indicated by the black dotted line in Figure 10 aligns well, particularly at 2θ = 27.3° and 31.8°, corresponding to the absorption peaks of NaCl, due to the buffer’s NaCl content before vacuum freeze-drying of the sample. Consistent with prior studies, typical diffraction peaks related to the crystalline structure of Se (2θ = 23.5° and 29.7°) were detected in ELNs [74,75], indicating the presence of crystalline selenium in ELNs. In contrast, for SeNPs and the three SeNPs-ELNs, the typically sharp and narrow selenium diffraction peaks were absent, especially in the range of 20° < 2θ < 30°. This suggests that after the formation of SeNPs-ELNs using ELNs as a template, the structural material of ELNs altered, and the selenium in the samples became amorphous [29]. The formation of amorphous particles might be due to the presence of proteins, nucleic acids, and lipids in ELNs, which contain chemical groups such as amino, carboxyl, and hydroxyl. These groups have high electron density or coordination capabilities, interacting with SeNPs, obstructing the growth of SeNPs, and thus leading to the formation of amorphous substances [33].

### 3.6. SEM Analysis

**Figure 11:**
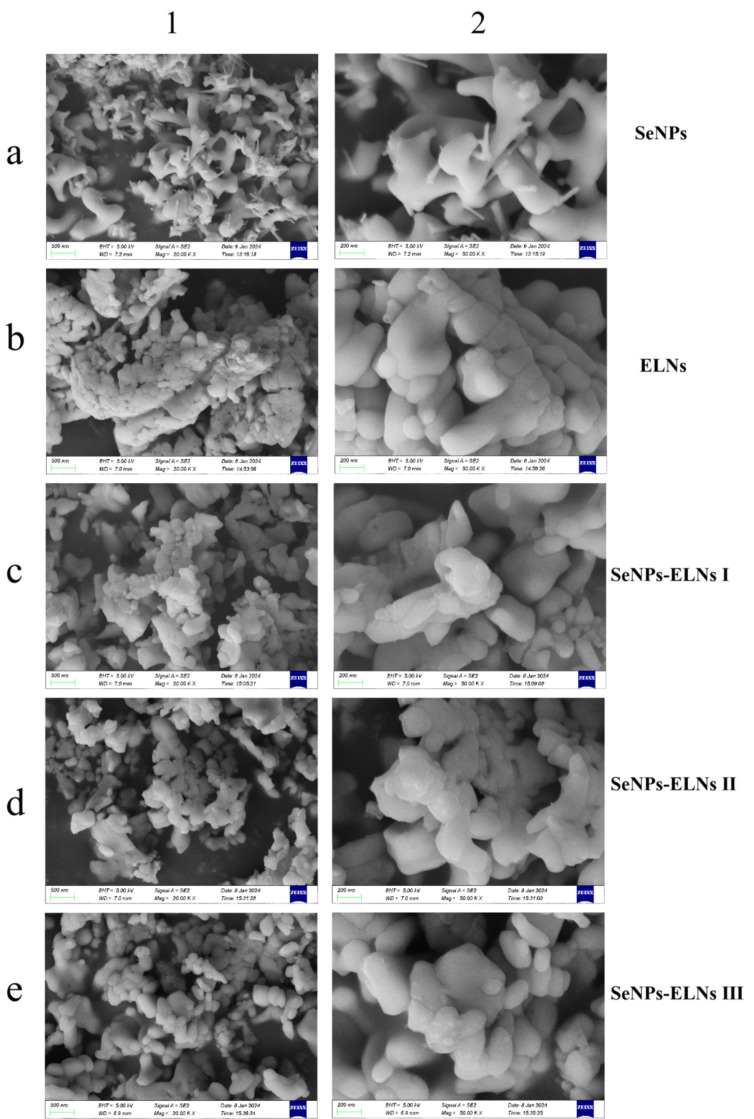
(**a**–**e**) correspond to SeNPs, ELNs, SeNPs-ELNs I, SeNPs-ELNs II, and SeNPs-ELNs III, respectively; (**1**,**2**) represent the scale bars 500 nm and 200 nm.

As observed in Figure 11a, SeNPs formed from the reaction between sodium selenite and ascorbate tend to cluster and exhibit sticky adhesion [69]. The SeNPs of Figure 11(a2) also show the uneven thickness of selenium lines, a result of the growth capacity being influenced by vacuum freeze-drying. These selenium lines are consistent with the morphology of the SeNPs shown in the TEM-EDS Mapping analysis in Figure 8(a1). The varied morphology of selenium lines and sticks also confirms the characteristics of broad SeNPs absorption peaks, poor symmetry, and uneven particle size distribution noted in the UV-visible spectrum (Figure 7). The particle dispersion of ELNs was very poor, with significant agglomeration, indicating that ELNs samples are not suited for freeze-drying. Similarly, vacuum freeze-drying significantly impacts the morphology of the three SeNPs-ELNs, leading to aggregation. Among the SeNPs-ELNs particles prepared by the three methods, those created using the SeNPs-ELNs III exhibited the best particle integrity.

### 3.7. Determination of Loading Amount Analysis

**Figure 12:**
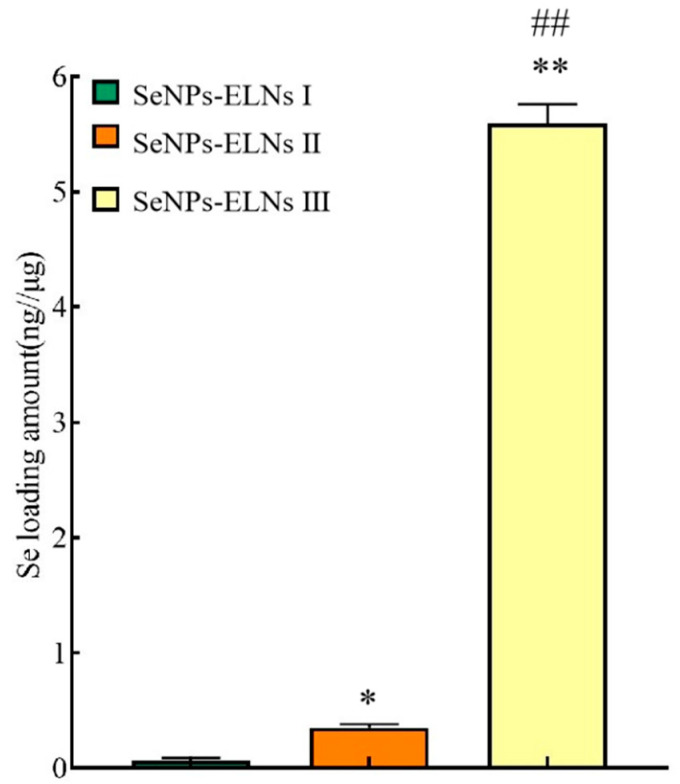
The SeNPs loading amount of different methods. Note: * denotes significant differences between SeNPs-ELNs I and other groups; * *p* < 0.05, ** *p* < 0.01. # denotes significant differences between SeNPs-ELNs II and SeNPs-ELNs III; ## *p* < 0.01.

In this study, one of the challenging tasks is loading SeNPs into exosomes effectively. Upon incorporation by various methods, we evaluated the SeNPs loading amount using hydride atomic fluorescence spectroscopy and the BCA protein method according to Section 2.11. As shown in Figure 12, determination of SeNPs loading results showed that SeNPs-ELNs III exhibited significantly higher selenium-loading (5.59 ± 0.167 ng/μg), followed by SeNPs-ELNs II (0.3501 ± 0.029 ng/μg) and SeNPs-ELNs I (0.062 ± 0.0256 ng/μg), respectively. During cargo-loaded exosome preparation, a higher concentration gradient is required in order for active components to diffuse into exosomes [76]. In addition, applied ultrasonic forces lead to transient reformation/deformation of exosomes, which, on the one hand, facilitate the diffusion of cargo, and on the other hand, disrupt exosome integrity [38]. The results obtained in our study might be the result of a low concentration gradient of SeNPs and disruption of exosomes by the ultrasonic washer.

### 3.8. TEM Morphology

**Figure 13:**
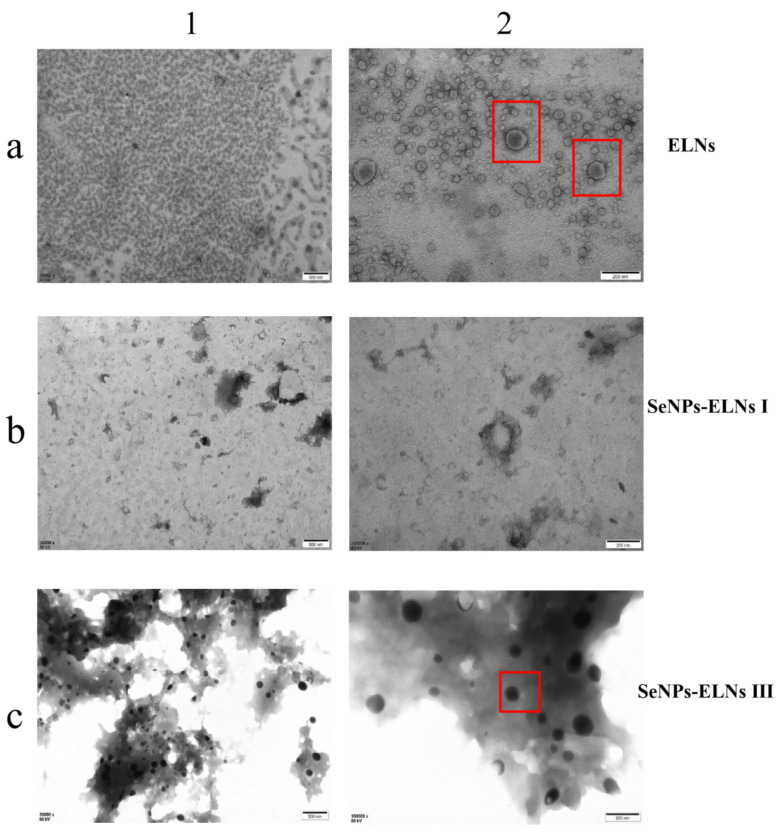
(**a**–**c**) correspond to ELNs, SeNPs-ELNs I, and SeNPs-ELNs III, respectively; (**1**,**2**) represent the scale bars 500 nm and 200 nm. The red box is used to highlight the clear structure of the nanoparticles.

To confirm the formation of SeNPs-ELNs, we examined the microstructure of ELNs and two SeNPs-ELNs samples prepared by an ultrasonic washer and ultrasonic cell fragmentation, using TEM imaging [77]. The morphologies of the three samples varied significantly (Figure 13). In line with other studies [40,78], exosomes from Cyperus beans displayed a heterogeneous, spherical concave structure with clear membrane margins, indicating intact and relatively pure exosomes. The concave structure of ELNs observed might result from exosome collapse during dehydration. The SeNPs-ELNs I appeared blurred under TEM, with a less distinct globular structure, likely due to disruption from the ultrasonic process [76].

Conversely, SeNPs-ELNs III presented a smooth, round surface, differing from unloaded exosomes. This diversity reflects the structural changes in exosomes upon composite formation. Characterization confirmed that substances isolated from Cyperus beans were exosomes and the successful introduction of SeNPs into ELNs. According to XRD, XPS, and visual assessments, the selenium in SeNP-ELNs was identified as red amorphous zero-valent selenium. Combined with FTIR, UV-Vis, TEM-EDS Mapping analysis, and hydride atomic fluorescence spectra, these results confirmed successful SeNP loading and indicated that ultrasonic cell fragmentation is the preferred loading method. SeNP-ELNs III prepared by this method were used for subsequent characterization, stability tests, cytotoxicity, and hemolysis experiments.

### 3.9. Particle Size, Zeta Potential, and Polydispersity Index

**Figure 14:**
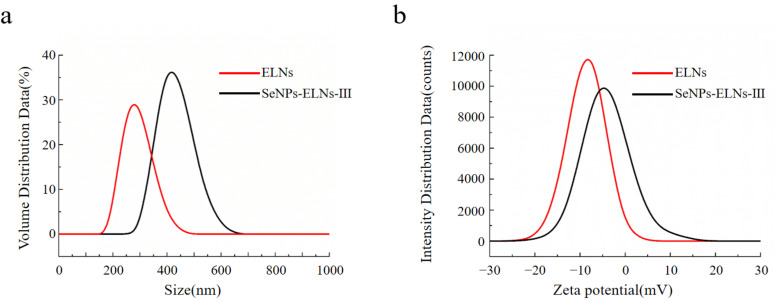
(**a**) Particle size distribution of ELNs and SeNPs-ELNs III, (**b**) Zeta potential of ELNs and SeNPs-ELNs III.

ELNs are known to play various biological roles, but their physicochemical properties remain less understood due to the complex interspecies composition [79]. The particle size, PDI, and zeta potential are essential for characterizing the physicochemical properties, colloidal stability, and bioavailability of ELNs [80]. As shown in Figure 14a, a unimodal particle size distribution was observed for both ELNs and SeNPs-ELNs III. The ELNs suspension was highly dispersed, with an average particle size of approximately 286.5 ± 6.11 nm and and a zeta potential of −8.37 ± 0.51 mV. Moreover, the suspension displayed higher PDI (0.826), indicating heterogeneity and reduced stability [81]. Although the size distribution in our study was larger than typically reported, the results are consistent with previous data on plant-derived exosome-related particles [82,83,84]. Upon formation of SeNPs-ELNs III, the PDI increased to 0.966, and a considerable shift of the particle size distribution towards larger size (431.17 ± 10.78 nm) and a significant (*p* < 0.05) reduction in absolute zeta potential (−4.1 ± 0.43 mV) were observed, implying the significant variation in composition of ELNs with the introduction of SeNPs. This aligns with previous studies indicating the impact of membrane phospholipids and lipid–protein interactions on the zeta potential of ELNs [85]. The synthesis method might disrupt the ELNs membrane, reducing electrostatic repulsion between particles and thus increasing particle size [86].

### 3.10. Temperature Stability Analysis

**Figure 15:**
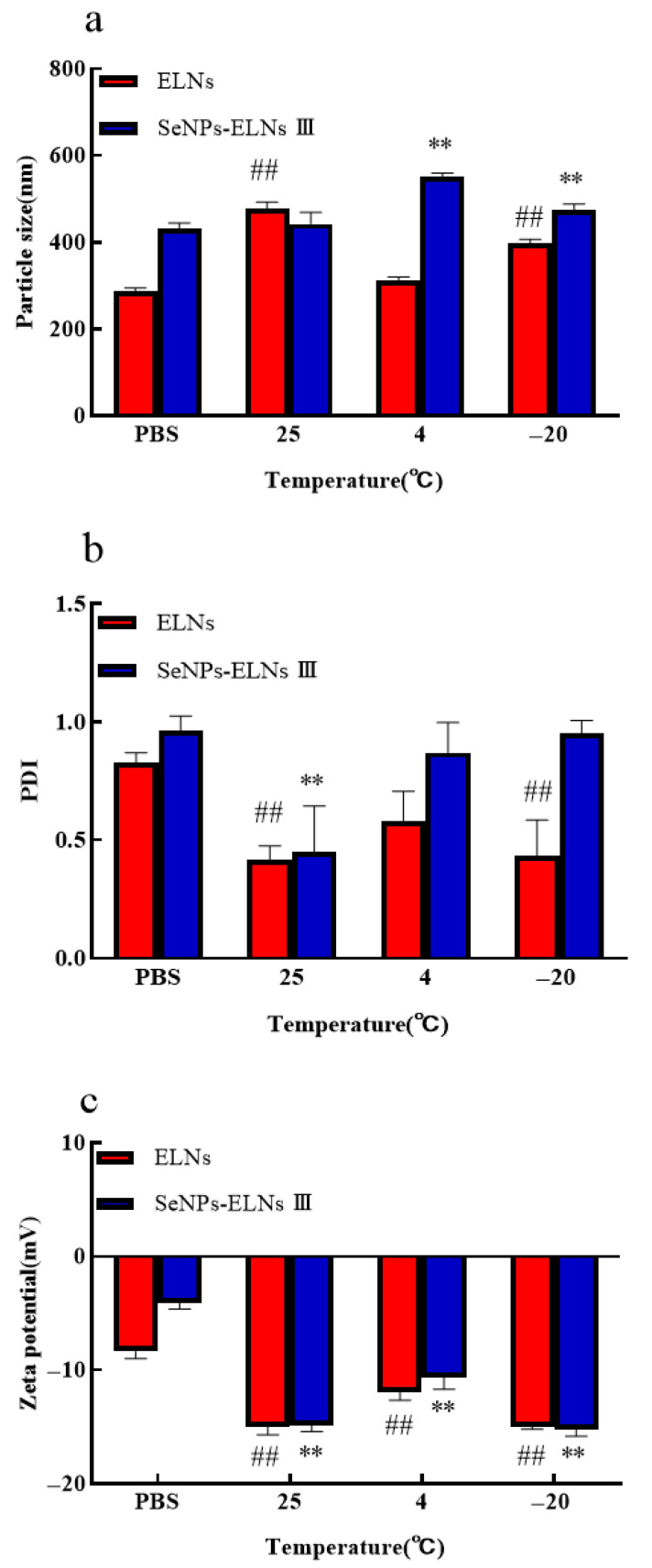
(**a**) Particle size vs. temperature; (**b**) PDI vs. temperature; (**c**) Zeta potential vs. temperature. Notes: Temperature Experiments (vs. PBS control): ##: highly significant (*p* < 0.01) differences for ELNs compared to PBS. **: highly significant (*p* < 0.01) differences for SeNPs-ELNs III compared to PBS.

Stability during storage is crucial for the nutritional or clinical application of nano-formulations. We evaluated the stability of ELNs and SeNPs-ELNs III stored at 25 °C, 4 °C, and −20 °C for 14 days by measuring particle size, PDI, and zeta potential (Figure 15a–c). The storage temperature significantly affected all measured parameters. Despite some contradictory findings [87], consistent with recent studies [88], both particle size and absolute zeta potential of ELNs generally increased at all tested temperatures. The smallest increase in particle size occurred at 4 °C (312 ± 6.7 nm), followed by −20 °C (398.2 ± 6.9 nm) and 25 °C (477.8 ± 11.6 nm) (Figure 15a). Similarly, upon comparison of the effects of different storage temperatures on the zeta potential of ELNs, a slight increase in charge was also observed at 4 °C (Figure 15c). As an indicator of particle homogeneity, changes in PDI during storage suggest varying stability levels at different temperatures. The PDI value of ELNs significantly decreased (*p* < 0.05) at 25 °C and −20 °C, indicating instability at these temperatures (Figure 15b). These changes might be due to particle aggregation or fusion at 25 °C and disruption of the ELNs lipid membrane caused by freezing at −20 °C.

In contrast, SeNPs-ELNs III showed a less significant increase in particle size at 25 °C (442.6 ± 21.7 nm) and more significant increases at 4 °C (551.4 ± 6.2 nm) and −20 °C (474.1 ± 11.6 nm) (Figure 15a). The determined zeta potential (Figure 15c) became significantly more negative after storage at different temperatures, with the greatest changes observed at 25 °C and −20 °C. Consistent with particle size results, the PDI of SeNPs-ELNs significantly decreased at 25 °C, while slight variations were observed at 4 °C and −20 °C (Figure 15b). These results suggest that lower temperatures may effectively enhance the stability of SeNPs-ELNs during storage.

### 3.11. pH Stability Analysis

**Figure 16:**
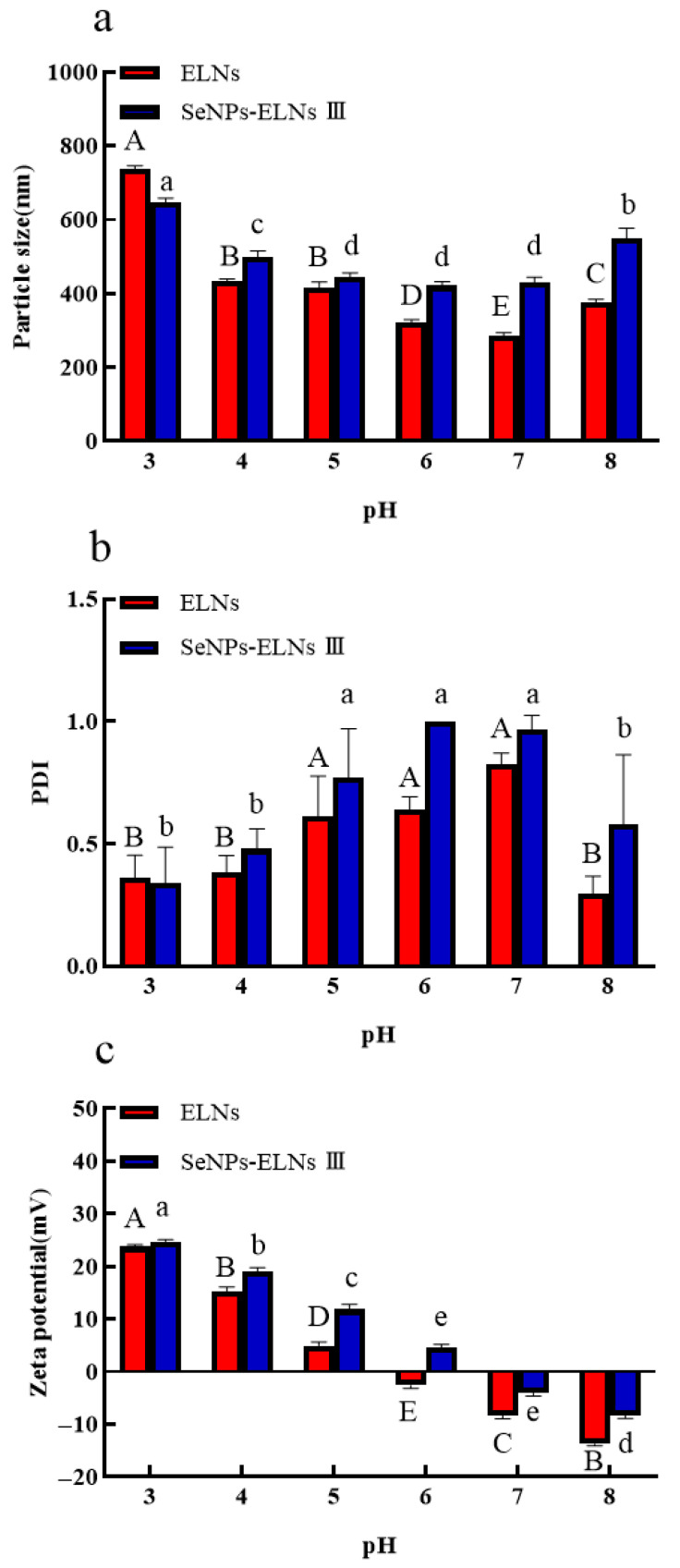
(**a**) Particle size vs. pH; (**b**) PDI vs. pH; (**c**) Zeta potential vs. pH. Notes: PH Experiments (multiple comparisons). Uppercase letters (A, B, C,…) denote significant differences (*p* < 0.05) among groups for ELNs. Groups sharing the same letter are not significantly different (*p* > 0.05). Lowercase letters (a, b, c,…) denote significant differences (*p* < 0.05) among groups for SeNPs-ELNs III. Groups sharing the same letter are not significantly different (*p* > 0.05).

The particle size of ELNs decreased with increasing pH up to pH 7, then increased slightly at pH 8. For SeNPs-ELNs III, an increase in pH from 3 to 4 significantly reduced the particle size, which then remained stable until pH 7 before increasing significantly at pH 8 (Figure 16a). At lower pH levels, both samples exhibited lower PDI values, suggesting that smaller exosomes were aggregating to form larger, more homogeneous structures. For both samples, an increase in pH led to a gradual increase in PDI up to pH 7, followed by a significant decrease at pH 8, with a more notable decrease (*p* < 0.05) observed for ELNs (Figure 16b).

To better understand surface interactions at different pH levels, we also investigated the effects of pH on zeta potential variation of ELNs and SeNPs-ELNs III. The results indicated that the zeta potential of both samples was highest at pH 3 and shifted gradually from positive to negative values across the pH spectrum, with the shift in ELNs occurring more rapidly than in SeNPs-ELNs III (Figure 16c). Exosomes typically possess negative charges, maintained by phospholipids and proteins in their membranes [85]. The surface charge could be changed by neutralizing anionic or cationic sites on their surfaces via excess amounts of protons (H^+^) under acidic conditions or excess base under basic conditions [89]. It has been reported that exosomes need an absolute zeta potential of at least 20–30 mV to remain stable [90]. In our study, the observed zeta potential was below this range, resulting in considerable pH-dependent changes.

### 3.12. Digestive Stability Analysis

**Figure 17:**
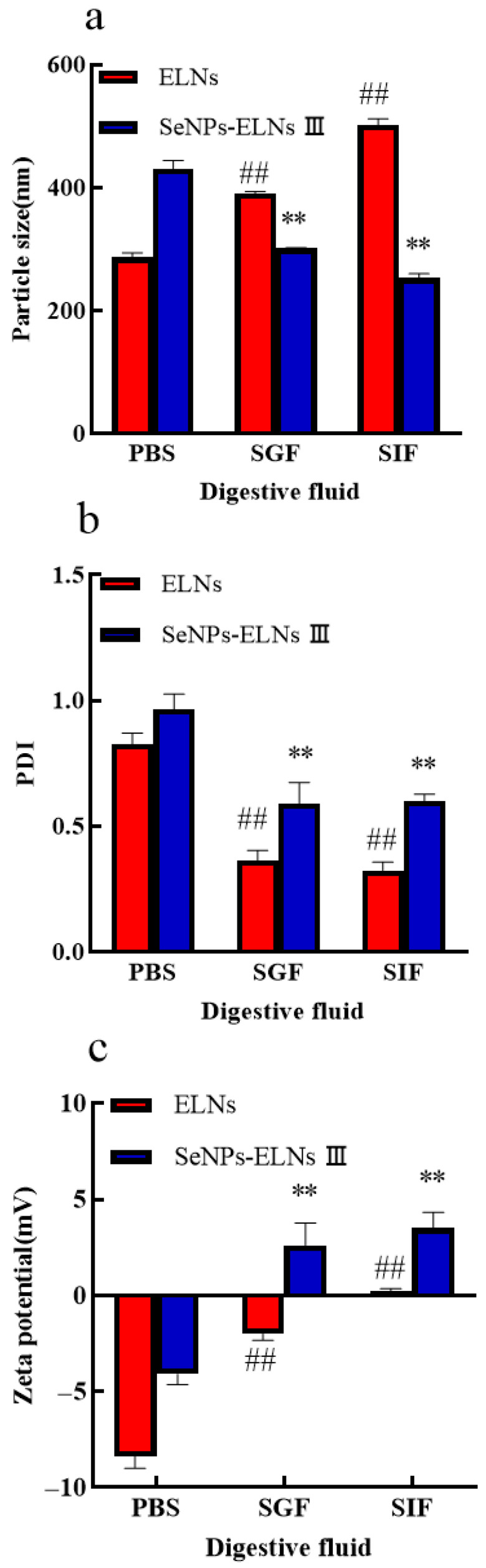
(**a**) Particle size in simulated gastrointestinal conditions; (**b**) PDI in simulated gastrointestinal conditions; (**c**) Zeta potential in simulated gastrointestinal conditions. Notes: Digestive Environment Experiments (vs. PBS control): ##: highly significant (*p* < 0.01) differences for ELNs compared to PBS. **: highly significant (*p* < 0.01) differences for SeNPs-ELNs III compared to PBS.

To assess the stability of the two samples, we examined the digestion behaviors of ELNs and SeNPs-ELNs III in PBS (control), SGF, and SIF by measuring particle size, PDI, and zeta potential. As shown in Figure 17a,b, during incubation in SGF for 2 h and in SIF for 7 h, ELNs experienced an increase in particle size and a decrease in PDI. We speculate that these changes may be due to two factors: the preferential digestion of smaller-sized exosomes and the aggregation of these exosomes due to a reduction in surface lipids, which results in fewer small-sized exosomes remaining in suspension. In addition, the zeta potential of ELNs shifted from a negative to a positive charge compared to the control (Figure 17c), further confirming the reduction in surface lipids during digestion. These findings align with those obtained from studies on ginger exosome digestion [91]. Interestingly, a decrease in particle size and shift in zeta potential from negative to positive were observed for SeNPs-ELNs III composite, demonstrating their breakdown during digestion. Compared to the control, PDI values for both ELNs and SeNPs-ELNs III significantly decreased after digestion, with a more pronounced decrease for ELNs, indicating a shift from heterogeneity to homogeneity during digestion. Moreover, both ELNs and SeNPs-ELNs III showed similar significant declines in PDI values, with ELNs exhibiting a lower PDI than SeNPs-ELNs III, suggesting greater resistance of SeNPs-ELNs III to digestive processes (Figure 17b). It is important to note that lipase is not present in SGF and SIF, and the phospholipid bilayer is not digested by pepsin and trypsin. Therefore, the digestive stability experiment only represents a portion of the phenomenon simulated in vitro and cannot fully replace the real digestion process of ELNs and SeNPs-ELNs III in vivo. This stability suggests potential for further development as selenium carriers—pending validation in complex biological systems.

### 3.13. In Vitro Cytotoxicity Study

**Figure 18:**
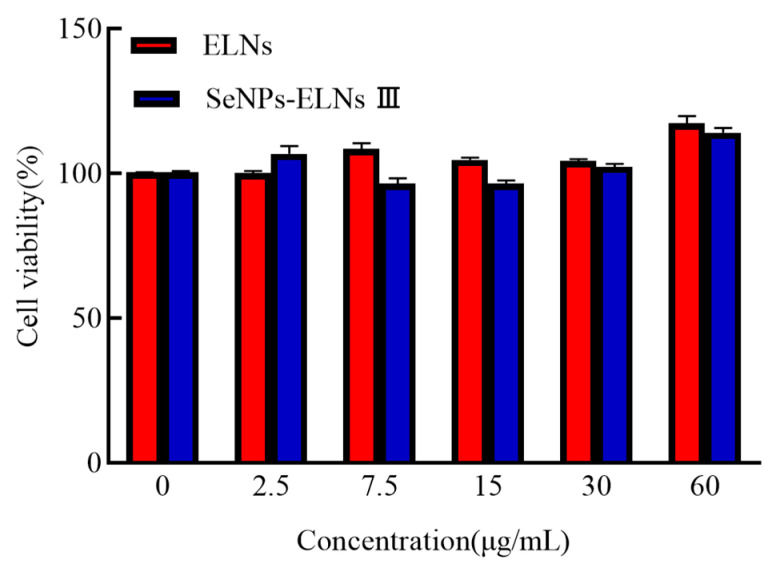
The LO_2_ cell viability of ELNs and SeNPs-ELNs III.

After exposure of LO_2_ cells to protein concentrations of 2.5, 7.5, 15, 30, and 60 μg/mL for 24 h, absorbance values at 450 nm were measured using the CCK-8 assay. Neither ELNs nor SeNPs-ELNs III exhibited cytotoxic effects within this concentration range (Figure 18), akin to a previous study on human RA fibroblasts exosomes [92].

### 3.14. Relative Erythrocyte Hemolysis Rate Analysis

**Figure 19:**
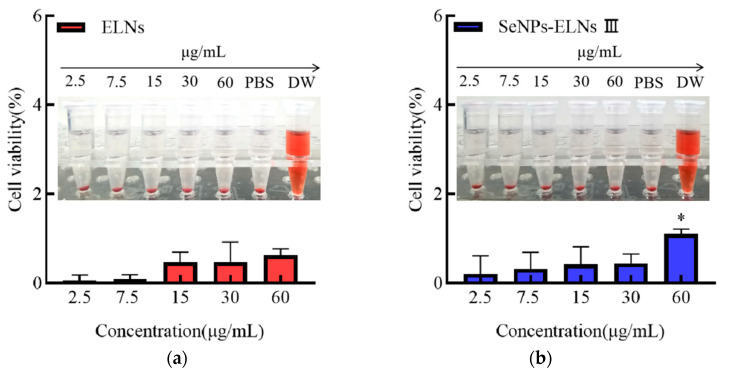
(**a**) Relative erythrocyte hemolysis rate of ELNs, (**b**) Relative erythrocyte hemolysis rate of SeNPs-ELNs III. Note: Significant differences compared to the 2.5 μg/mL protein concentration group. * denote significant (*p* < 0.05) differences vs. SeNPs-ELNs III.

As depicted in Figure 19, the relative erythrocyte hemolysis rates for ELNs and SeNPs-ELNs III at protein concentrations of 2.5, 7.5, 15, 30, and 60 μg/mL were <5%. This demonstrates good erythrocyte compatibility for both samples, similar to earlier findings where HTPP-Exo-M1–8 showed no noticeable hemolytic effects on mouse blood [64].

## 4. Conclusions

In this study, ELNs were successfully extracted from Cyperus beans, and ultrasonic cell fragmentation was demonstrated to be an effective method for incorporating exosomes with SeNPs. The stability of SeNPs-loaded exosomes was influenced by storage temperatures, with high temperatures or freezing significantly destabilizing the exosomes, leading to aggregation and the formation of homogeneous exosomes. Additionally, exosomes’ stability was found to be pH-dependent, with highly acidic or basic suspension conditions contributing to improved stability. Furthermore, the digestion test results in vitro indicated that the SeNPs-loaded exosomes exhibited resistance to digestion by both SGF and SIF in the study, suggesting that these exosomes could serve as promising carriers for selenium. Moreover, neither the ELNs nor the SeNPs-ELNs III exhibited cytotoxicity toward LO_2_ cells, and relative erythrocyte hemolysis remained low at protein concentrations of 2.5, 7.5, 15, 30, and 60 μg/mL.

## 5. Patents

Xinjiang Agricultural University. “A selenium-enriched oilseed soybean exosome and its preparation method and application” CN117645969A.2024-03-05.

## Figures and Tables

**Table 1:** Element content.

Sample	Element	Atom(%)
SeNPs	N	4.85
	O	11.68
	Se	83.46
ELNs	N	26.43
	O	73.57
	Se	ND
SeNPs-ELNs I	N	0.79
	O	99.21
	Se	ND
SeNPs-ELNs II	N	44.37
	O	55.46
	Se	0.17
SeNPs-ELNs III	N	4.48
	O	93.8
	Se	1.72

## Data Availability

The original contributions presented in the study are included in the article/Supplementary Material, further inquiries can be directed to the corresponding author.

## Supplementary Materials

The following supporting information can be downloaded at: https://www.mdpi.com/article/10.3390/foods14152724/s1, Figure S1. EDS elemental mapping images: (a) SeNPs, (b) ELNs, (c) SeNPs-ELNs I, (d) SeNPs-ELNs II, (e) SeNPs-ELNs III; Figure S2. the original drawing of Figure 10 about ELNs, SeNPs, SeNPs-ELNs I, SeNPs-ELNs II, SeNPs-ELNs III.

## Abbreviations

The following abbreviations are used in this manuscript:

SeNPs	Selenium nanoparticles
ELNs	Exosome-like nanoparticles
SeNPs-ELNs	SeNPs-loaded exosomes
SeNPs-ELNs I	The combination of ELNs and SeNPs prepared by an ultrasonic washer
SeNPs-ELNs II	The combination of ELNs and SeNPs prepared by incubation
SeNPs-ELNs III	The combination of ELNs and SeNPs prepared by an ultrasonic cell fragmentation instrument
Na_2_SeO_3_	Sodium selenite
FTIR	Fourier transform infrared spectroscopy
UV-Vis	UV-visible spectrum
TEM-EDS	Transmission electron microscopy and energy dispersive X-ray spectroscopy
XPS	X-Ray photoelectron spectroscopy
XRD	X-Ray diffraction
DLS	Dynamic light scattering
PEG	Polyethylene glycol
PDI	Polydispersity index
SEM	Scanning electron microscope
LC	Load volume
BCA	Bicinchoninic acid
BSA	Bovine serum albumin
TEM	Transmission electron microscopy
PBS	Phosphate-buffered saline
CCK-8	Cell Counting Kit-8
ANOVA	Analysis of variance
SGF	Simulated gastric fluid
SIF	Simulated intestinal fluid
