# Supplementary material for: Preparation and Characterization of Cyperus-Derived Exosomes Loaded with Selenium Nanoparticles for Selenium Delivery Based on Exosome Protein Quantitation
Source: Foods. 2025 Aug 4;14(15):2724. doi: 10.3390/foods14152724 (PMC12346761; doi:10.3390/foods14152724)
Supplement: Supplementary file 1 [file foods-14-02724-s001.zip › foods-3761888-supplementary.pdf]

Supplementary Figure legends

Supplementary Figure S1. EDS elemental mapping images: (a) SeNPs, (b) ELNs, (c) SeNPs-ELNs I, (d) SeNPs-ELNs II, (e) SeNPs-ELNs III.

Supplementary Figure S2. the original drawing of Fig. 10 about ELNs, SeNPs, SeNPs-ELNs I, SeNPs-ELNs II, SeNPs-ELNs III.

Supplementary Figure

Supplementary Figure S1

a

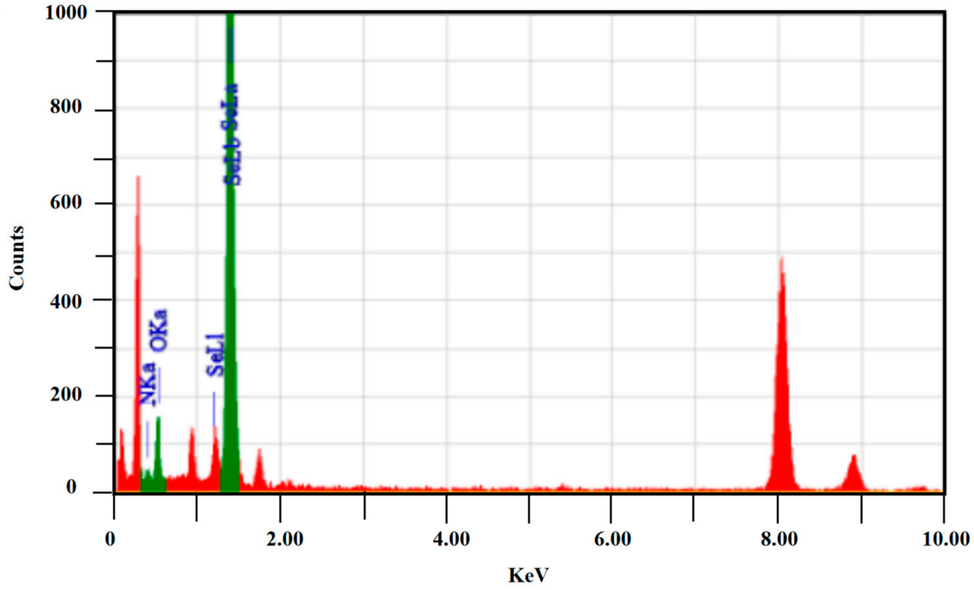

b

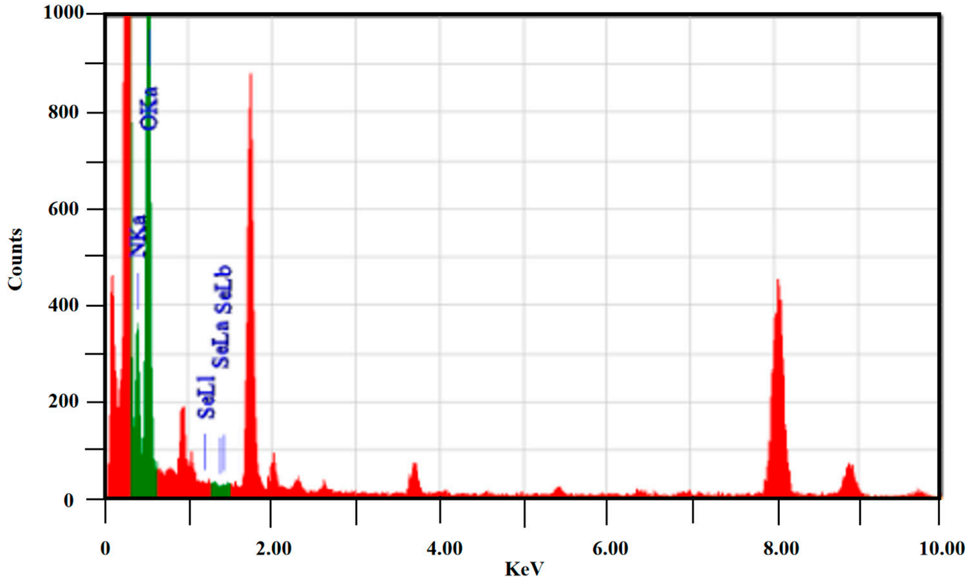

c

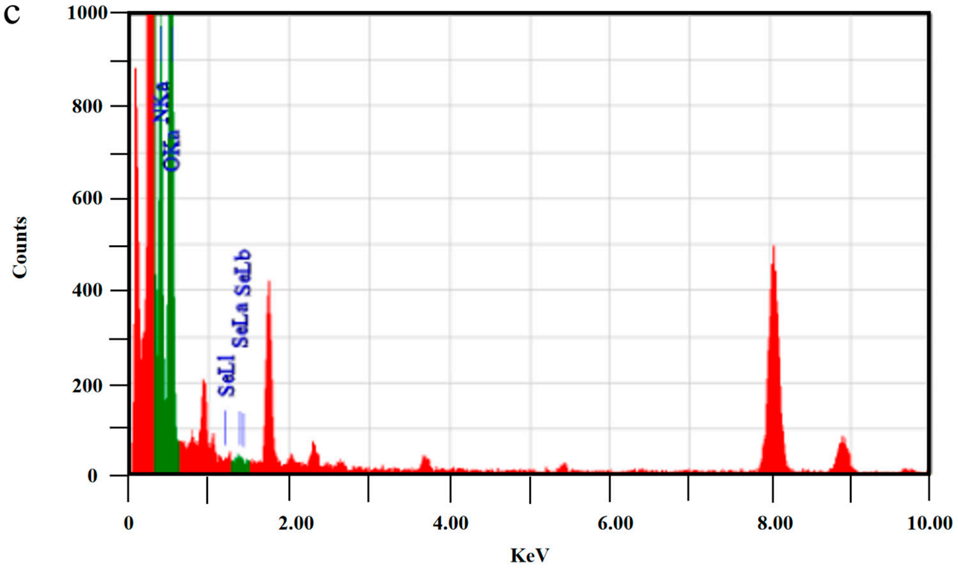

Counts
KeV
NKa
OKa
SeLl
SeLa
SeLb
0
200
400
600
800
1000
2.00
4.00
6.00
8.00
10.00

d

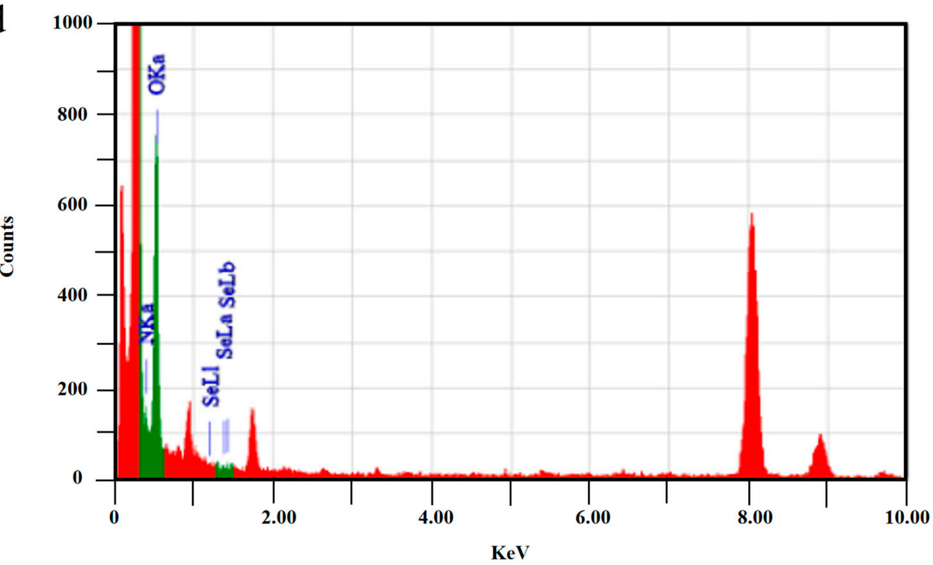

Counts
KeV
OKa
NKa
SeLl
SeLa
SeLb
0
200
400
600
800
1000
2.00
4.00
6.00
8.00
10.00

e

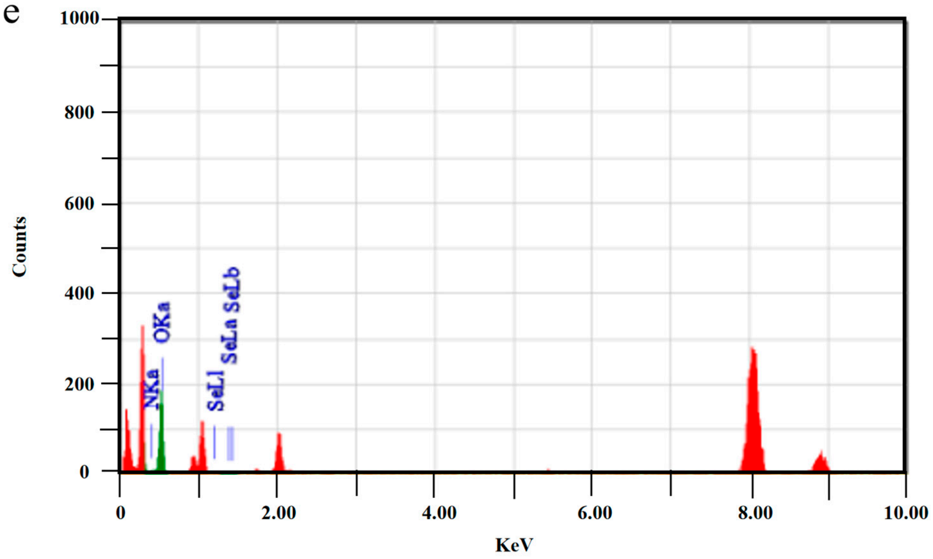

Counts
KeV
NKa
OKa
SeLl
SeLa
SeLb
0
200
400
600
800
1000
2.00
4.00
6.00
8.00
10.00

Supplementary Figure S2

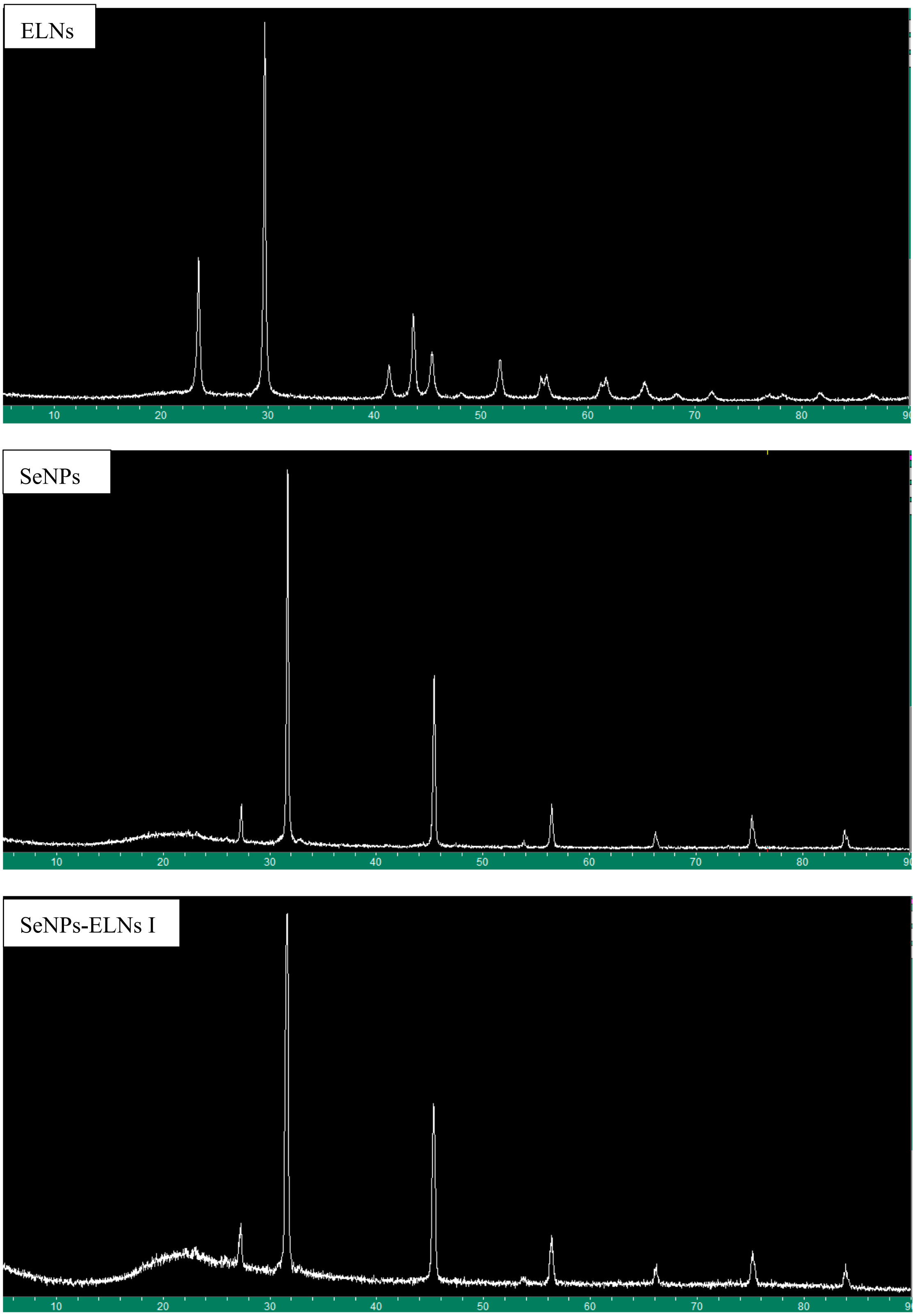

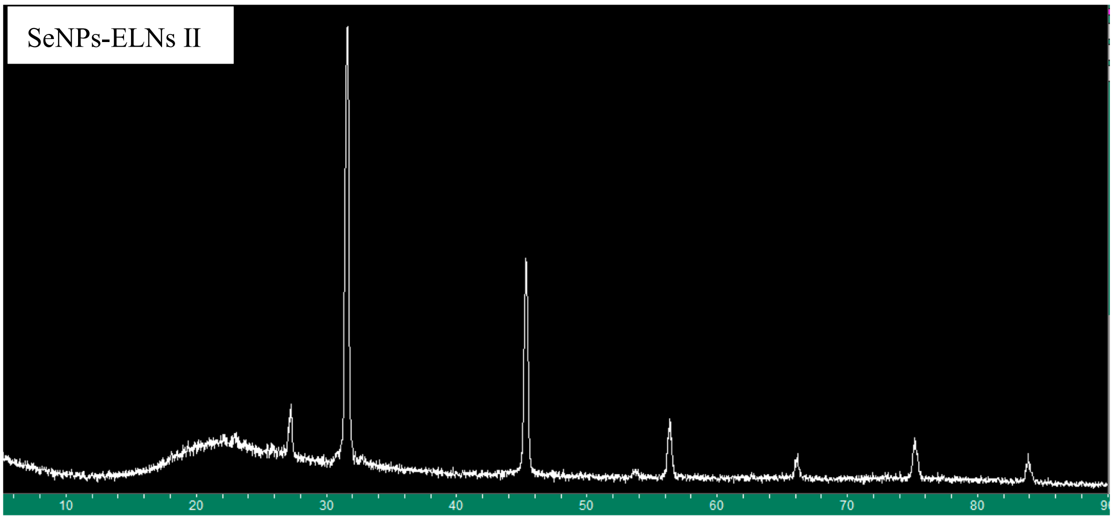
SeNPs-ELNs II
10
20
30
40
50
60
70
80

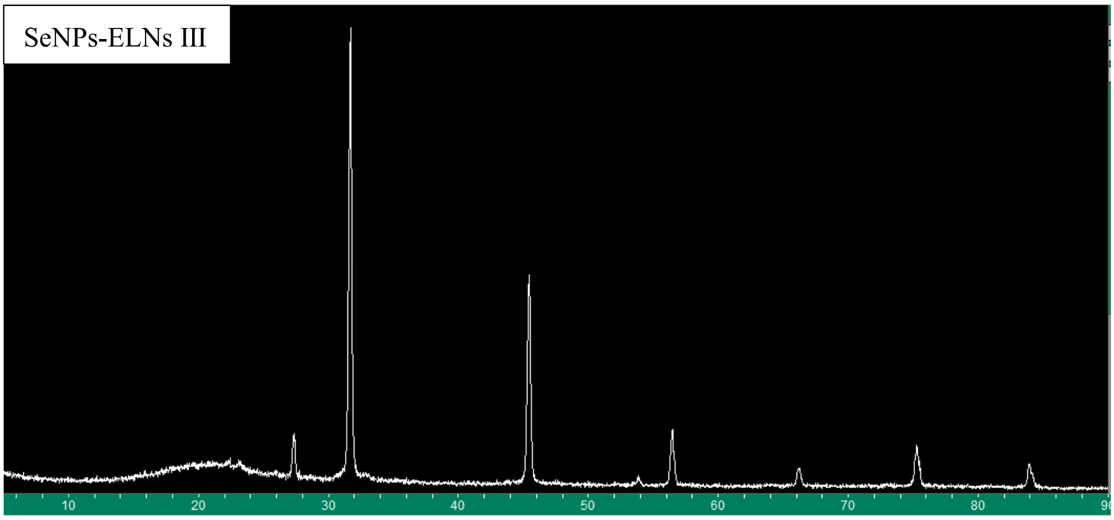
SeNPs-ELNs III
10
20
30
40
50
60
70
80
